# Inducible promoters of bacterial microcompartments improve the CRISPR/Cas9 tools for efficient metabolic engineering of *Clostridium ljungdahlii*

**DOI:** 10.1128/aem.02183-24

**Published:** 2025-03-26

**Authors:** Jun-Zhe Zhang, Yu-Zhen Li, Zhi-Ning Xi, Yue Zhang, Zi-Yong Liu, Xiao-Qing Ma, Fu-Li Li

**Affiliations:** 1 State Key Laboratory of Photoelectric Conversion and Utilization of Solar Energy, Qingdao Institute of Bioenergy and Bioprocess Technology, Chinese Academy of Sciences 85437 , Qingdao, China; 2 Shandong C1 Refinery Engineering Research Center, Qingdao New Energy Shandong Laboratory, Qingdao Institute of Bioenergy and Bioprocess Technology, Chinese Academy of Sciences 85437 , Qingdao, China; 3 University of Chinese Academy of Sciences 521953, Beijing, China; 4 Haide College, Ocean University of China 12591, Qingdao, China; 5 Shandong Energy Institute, Qingdao, China; Chalmers tekniska hogskola AB, Gothenburg, Sweden

**Keywords:** genetic tools, metabolic engineering, *Clostridium ljungdahlii*, inducible promoter

## Abstract

*Clostridium ljungdahlii*, as a model acetogen strain, represents a novel platform for biotechnological production for CO_2_ fixation. The genome of *C. ljungdahlii* harbors two gene loci associated with glycyl radical enzyme-associated microcompartments (GRMs), which are predicted to play essential roles in choline and 1,2-propanediol (1,2-PD) metabolism. This study validated the functions of these GRM loci and identified two inducible promoters, of which P_choline1_ was induced by choline, while P_1,2-PD_ was induced by 1,2-PD. Subsequently, the highly expressed P_1,2-PD_ and tightly controlled P_choline1_ were applied to improve CRISPR/Cas9 gene editing tools. Specifically, P_1,2-PD_ was used to develop a highly efficient gene knockout tool based on an all-in-one plasmid, achieving 100% deletion efficiency for multiple genes, including *pyrE*, *pduS*, *aor2*, and *eutT*. On the other hand, the *cas9* gene was integrated downstream of P_choline1_ into the genome. The integrated *cas9* efficiently mediated gene editing in *C. ljungdahlii* by introducing plasmids containing a gRNA cassette along with the relevant homology arms. This was exemplified by the construction of the *Δbdh::pdc* strain, where the 2,3-butanediol dehydrogenase gene was replaced with a pyruvate decarboxylase gene from *Zymomonas mobilis* and the 3-HB Syn KI strain, in which an artificial 3-hydroxybutyric acid synthesis pathway was inserted into the genome. This study highlights the effectiveness and convenience of the inducible CRISPR/Cas9 gene editing systems, thereby enriching the CRISPR/Cas toolkit in acetogens.

**IMPORTANCE:**

A CRISPR/Cas9 genetic tool controlled by a constitutive promoter has been developed for precise gene deletion in *Clostridium ljungdahlii*. However, its efficiency was hindered by the toxicity resulting from the constitutive expression of cas9 and the large plasmids, leading to a low overall success rate. Inducible promoters, which allow for the transcription of target genes to be switched on and off in the presence or absence of inducers, have a broad range of applications. In this study, we identify two inducible promoters and apply them to enhance the CRISPR/Cas9 tools. The improved CRISPR/Cas9 tools facilitate gene editing with high efficiency, potentially playing significant roles in advancing genetic research and metabolic engineering of *C. ljungdahlii*.

## INTRODUCTION

Acetogenic bacteria are anaerobes that employ the Wood–Ljungdahl pathway to fix CO_2_ for the production of acetyl-CoA and subsequently for biocommodities and biofuels such as acetate, ethanol, and 2,3-butanediol using H_2_ or CO as energy sources (1, 2). *Clostridium ljungdahlii* is a model acetogen strain that was isolated from chicken yard waste for its ability to produce ethanol from syngas (mainly a mixture of CO, H_2_, and CO_2_). It is one of the most versatile acetogens with respect to substrate utilization (3, 4). In addition to acetate, *C. ljungdahlii* produces large amounts of ethanol as well as smaller amounts of 2,3-butanediol and lactate, indicative of its promising potential in industrial applications (5). With the rapid development of genetic tools for molecular biological manipulation, *C. ljungdahlii* is now a novel platform for biotechnological production based on C1 gases (CO_2_ and CO) (6, 7).

In recent years, the clustered regularly interspaced short palindromic repeats (CRISPR)/CRISPR-associated proteins (Cas) gene editing system has become a widely utilized method for precise and scarless gene editing (8, 9). Huang et al. have first established a CRISPR/Cas9 gene editing tool in *C. ljungdahlii* (10). The system includes a single-guide RNA (sgRNA) and *cas9* expression box, as well as an editing template for homologous recombination. The constitutive promoter, P_thl_ from *Clostridium acetobutylicum*, was utilized to regulate the expression of the Cas9 nuclease. This system has achieved an efficiency of over 50% in precise gene deletion. However, constitutive expression of *cas9* is generally toxic for both the *Escherichia coli* donor and the acetogen target, resulting in a low overall number of positive outcomes. It was shown that the *cas9* gene on the vectors was predisposed to mutation in *E. coli*, leading to the production of a truncated Cas9 protein. The truncated Cas9 was most likely a “nickase” variant of Cas9, producing single-strand nicks that appeared to be less toxic than the double-strand breaks induced by native Cas9 (11).

Employing inducible promoters to tightly control the expression of *cas9* is an alternative strategy. Since several attempts to introduce a plasmid constitutively expressing *cas9* into *Clostridium autoethanogenum* with sgRNA plasmids were not successful, a tetracycline-inducible promoter was used to regulate the expression of *cas9* (12). This system with an enhanced variant of the tetracycline-inducible promoter controlling the expression of *cas9* achieved an editing efficiency of over 50% in *C. autoethanogenum*. Recently, a theophylline-responsive riboswitch was reported to efficiently control the expression of *cas9* at the translational level in several non-acetogenic clostridia (13). It was later shown to function effectively for the generation of mutants in *C. autoethanogenum* (14).

Additionally, the size of the plasmids for the CRISPR/Cas system is often an issue as well. The plasmid that contains both the *cas9* gene and the editing template generally has a size over 10 kb, resulting in low transformation efficiencies. This issue can be avoided by using a two-plasmid system, in which one plasmid contains the *cas9* gene, and the other plasmid expresses the guide RNA (gRNA) containing homology arms (HAs) that serve as the DNA editing template (12). Besides the aforementioned strategies, the endogenous CRISPR/Cas systems can also be adopted for gene editing. Acetogens such as *Acetobacterium woodii* and *C. autoethanogenum* possess endogenous Type I-B CRISPR systems, and these systems have been successfully employed for in-frame deletion of genes in these organisms (15). However, no endogenous CRISPR system is present in *C. ljungdahlii* DSM 13528, although it is closely related with C. *autoethanogenum* (16). Hence, integration of *cas9* into the genome under the tight control of an inducible promoter may be an alternative way to optimize the CRISPR/Cas tool. This strategy has been successfully employed in *C. acetobutylicum*, where the *cas9* gene was integrated into the genome under the control of the xylose inducible system from *Clostridium difficile* (17).

Bacterial microcompartments (BMCs) are proteinaceous organelles involved in both anabolic and catabolic processes (18, 19). The known catabolic BMCs, metabolosomes, widely occur in bacteria and often play a role in the breakdown of various organic compounds such as propanediol, choline, and ethanolamine (18, 20, 21). The metabolosomes share a common biochemical core, including a signature enzyme that degrades a corresponding signature substrate to generate an aldehyde and a series of aldehyde-processing enzymes (22). This enzymatic core is encapsulated by an outer protein shell to form polygonal structures, composed of three types of proteins, hexameric proteins (BMC-H), trimeric proteins (BMC-T), and pentameric proteins (BMC-P). The genes encoding the subcellular structures and their cargo proteins are often organized as superloci and typically are expressed only in the presence of their signature substrates, suggesting the presence of inducible regulatory systems within the gene loci.

The genome of *C. ljungdahlii* contains two predicted BMC loci, which are predicted to play roles in choline and 1,2-propanediol (1,2-PD) metabolism (22, 23). This study presents the inducible assembly of these two BMCs and identifies two inducible promoters responsive to their respective signature substrates within the BMC loci. Furthermore, the two inducible promoters are applied to improve the CRISPR/Cas9 gene editing tool for the efficient metabolic engineering of *C. ljungdahlii*.

## RESULTS AND DISCUSSION

### The assembly of BMCs in *C. ljungdahlii* was induced in the presence of choline and 1,2-PD

The existence of two BMC loci in the *C. ljungdahlii* genome was initially suggested by bioinformatic analyses, both of which contain genes that encode regulatory proteins, BMC shell proteins, and enzymes that are involved in signature substrate metabolism (Table 1) (20, 22). The signature enzymes encoded in these loci belong to the glycyl radical enzyme (GRE) family and are predicted to degrade choline and 1,2-PD. Consequently, the two glycyl radical enzyme-associated microcompartments (GRMs) are further classified as GRM1 and GRM3. Moreover, as the glycyl radical (Gly·) of GRE is produced post-translationally, a GRE-specific activase, known as activating enzyme (AE), is encoded within each BMC cluster (Table 1).

**TABLE 1:** Gene organization of the two BMC loci

GRM1			GRM3		
Gene locus	Predicted function	Log_2_(fold change) (choline vs fructose)	Gene locus	Predicted function	Log_2_(fold change) (1,2-PD vs fructose)
CLJU_RS19540	Alcohol dehydrogenase	6.0 ± 1.1	CLJU_RS05805	GRE3 (1,2-PD dehydratase)	9.7 ± 1.5
CLJU_RS19545	Histidine kinase	0.4 ± 0.8	CLJU_RS05810	AE3	8.8 ± 1.6
CLJU_RS19550	Response regulator	0.2 ± 0.8	CLJU_RS05815	Response regulator	0.8 ± 2.9
CLJU_RS19555	BMC shell protein BMC-H	5.7 ± 1.0	CLJU_RS05820	Histidine kinase	2.6 ± 2.8
CLJU_RS19560	BMC shell protein BMC-H	5.7 ± 1.0	CLJU_RS05825	BMC shell protein (PduB)	10.5 ± 0.2
CLJU_RS19565	Phosphate acyltransferase	6.1 ± 0.8	CLJU_RS05830	Alcohol dehydrogenase	10.8 ± 0.3
CLJU_RS19570	Acetaldehyde dehydrogenase	5.6 ± 0.4	CLJU_RS05835	Aquaporin	9.3 ± 0.3
CLJU_RS19575	BMC shell protein BMC-H	5.4 ± 0.7	CLJU_RS05840	EutT function unknown	9.5 ± 0.6
CLJU_RS19580	Acetate kinase	7.6 ± 0.9	CLJU_RS05845	EutJ function unknown	7.7 ± 1.6
CLJU_RS19585	BMC shell protein BMC-T	6.0 ± 0.9	CLJU_RS05850	BMC shell protein BMC-H	7.0 ± 2.0
CLJU_RS19590	PduS (function unknown)	6.2 ± 0.9	CLJU_RS05855	BMC shell protein BMC-P	8.1 ± 1.6
CLJU_RS19595	BMC shell protein BMC-P	5.6 ± 0.4	CLJU_RS05860	Acetate kinase	8.7 ± 1.7
CLJU_RS19600	BMC shell protein BMC-H	4.4 ± 0.1	CLJU_RS05865	BMC shell protein BMC-H	6.5 ± 1.5
CLJU_RS19605	EutJ function unknown	5.3 ± 0.3	CLJU_RS05870	Acetaldehyde dehydrogenase	6.5 ± 0.9
CLJU_RS19610	EutT function unknown	6.9 ± 1.5	CLJU_RS05875	Phosphate propanoyltransferase	6.7 ± 0.5
CLJU_RS19615	AE1	6.7 ± 1.3	CLJU_RS05880	BMC shell protein BMC-H	6.5 ± 0.8
CLJU_RS19620	GRE1 (choline lyase)	7.9 ± 0.1	CLJU_RS05885	BMC shell protein BMC-H	6.5 ± 0.8
CLJU_RS19625	Aldehyde dehydrogenase	7.2 ± 0.9			
CLJU_RS19630	BMC shell protein BMC-H	7.5 ± 1.3			
CLJU_RS19635	BMC shell protein BMC-H	8.3 ± 0.9			

To validate the assembly and functionality of the two GRMs, the *C. ljungdahlii* strain was cultivated on a mixed carbon source, consisting of fructose and choline or 1,2-PD. As expected, choline and 1,2-PD were utilized as carbon sources, resulting in the accumulation of relevant acids and alcohols (Fig. S1A and B). Meanwhile, transmission electron microscopy of thin sections from *C. ljungdahlii* cells cultivated with choline or 1,2-PD revealed the presence of polyhedral structures, which were presumed to be BMCs (Fig. S1C and D). Concurrently, a significant upregulation of the genes within GRM1 loci was observed in the presence of choline, whereas the expression of the genes within GRM3 loci was notably induced upon exposure to 1,2-PD (Table 1).

### The GRM clusters contain three putative inducible promoters

**Fig 1:**
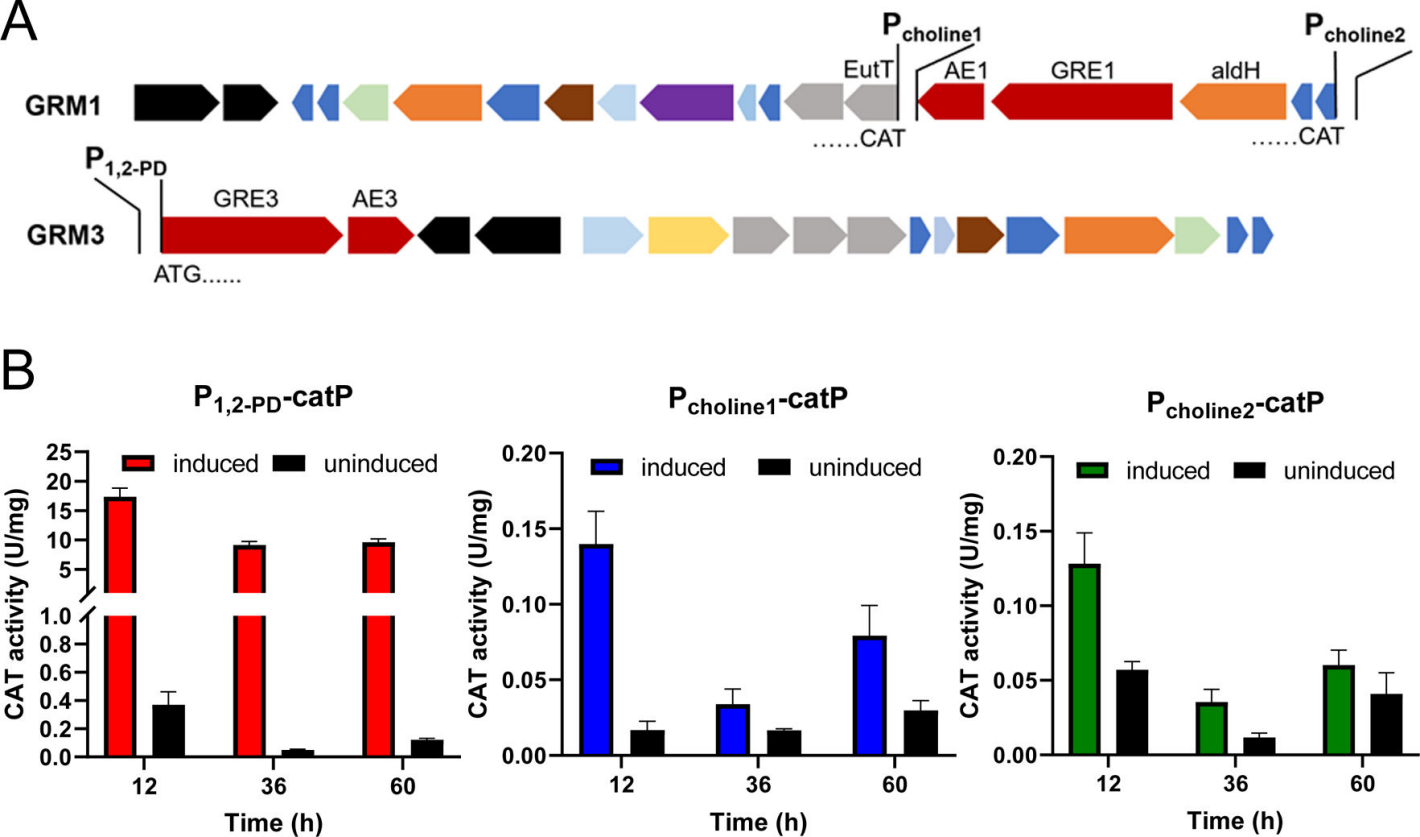
Identification of inducible promoters in the GRM loci. (A) The position of predicted inducible promoters. (B) The activity of the CatP reporter under control of the three predicted inducible promoters. The CAT activity was indicated in units/mg protein.

The assembly of GRMs was induced under choline or 1,2-PD exposure, suggesting that the expression of GRM genes is likely modulated by inducible promoters responsive to these compounds. Given the pronounced interest in inducible promoters for synthetic biology applications, we, therefore, investigated the inducible promoters within the GRM gene clusters. An examination of the intergenic sequences in the two GRM gene clusters uncovered three putative σ54 promoters. The predicted 1,2-PD-responsive promoter, located within the GRM3 cluster, spans 242 base pairs (bp) upstream of the GRE3 (CLJU_RS05805) start codon. Two choline-responsive promoter candidates were found: one lies between *AE1* (CLJU_RS19615) and *eutT* (CLJU_RS19610), spanning 316 bp upstream of eutT’s start codon, while the other extends 406 bp upstream of CLJU_RS19635 (Fig. 1A).

To access the transcriptional activity of the predicted promoters, namely, P_1,2-PD_, P_choline1_, and P_choline2_, PCR-amplified fragments of the promoters were cloned into the pMTL82254 vector (Table 2), respectively, upstream of the chloramphenicol acetyltransferase gene (*catP*) (24). The native ribosomal-binding site (RBS) and the spacer between the RBS and the start codon were retained for each promoter construct. The expression of *catP* was determined under both non-inducing and inducing conditions. As depicted in Fig. 1B, induction with 3 g/L 1,2-PD significantly enhanced the expression of *catP* under the control of P_1,2-PD_ by over 40 times. When exposed to 5 g/L choline, the activity of CatP under the regulation of P_choline1_ and P_choline2_ increased by eight and three times, respectively. Notably, both choline-responsive promoters exhibited lower basal expression levels compared to P_1,2-PD_. Even under inducing conditions, their expression activity remained below the basal expression level of P_1,2-PD_. Moreover, the P_1,2-PD_ consistently demonstrated induced activity throughout the cultivation period, in contrast to P_choline1_ and P_choline2_, which showed significant induction only during the initial 12 hours, in accordance with the choline consumption pattern (Fig. S1).

**TABLE 2:** Plasmids and strains

Strain or plasmid	Genotype	Source/reference
Strains		
DH5α	*Escherichia coli* K-12 cloning strain	
DSM 13528	Wild-type *Clostridium ljungdahlii* strain	DSMZ
CL-ΔpyrE	DSM13528 *ΔpyrE* (CLJU_RS17560)	This study
CL-ΔpduS	DSM13528 *ΔpduS* (CLJU_RS19590)	This study
CL-Δaor2	DSM13528 *Δaor2* (CLJU_RS09915)	This study
CL-ΔeutT	DSM13528 *ΔeutT* (CLJU_RS19610)	This study
CL-cas9KI	DSM13528::*cas9*	This study
CL-Δbdh::pdc	DSM13528::*cas9 Δbdh::pdc*	This study
CL-3HB Syn KI	DSM13528::*cas9 ΔP_3hbdh_::CA_thl, CA_ctfA, CA_ctfB*	This study
Plasmids		
pMTL82254	pBP1 *ori, ermB, ColE1 +tra, catP* reporter	(24)
pMTL83151	pCB102 *ori, catP, ColE1 +tra*	(24)
pMTLcas-pyrE	pCB102 *ori, catP, ColE1 +tra, P_thl_-cas9, P_araE_-sgRNA, pyrE* homologous arms	(10)
pMTL82254-P_1,2-PD_	pBP1 *ori, ermB, ColE1 +tra, P_1,2-PD_-catP* reporter	This study
pMTL82254-P_choline1_	pBP1 *ori, ermB, ColE1 +tra, P_choline1_-catP* reporter	This study
pMTL82254-P_choline2_	pBP1 *ori, ermB, ColE1 +tra, P_choline2_-catP* reporter	This study
pMTLP_1,2-PD_cas-ΔpyrE	pCB102 *ori, catP, ColE1 +tra, P_1,2-PD_-Cas9, P_araE_-sgRNA, pyrE* homologous arms	This study
pMTLP_1,2-PD_cas-ΔpduS	pCB102 *ori, catP, ColE1 +tra, P_1,2-PD_-Cas9, P_araE_-sgRNA, pduS* homologous arms	This study
pMTLP_1,2-PD_cas-Δaor2	pCB102 *ori, catP, ColE1 +tra, P_1,2-PD_-Cas9, P_araE_-sgRNA, aor2* homologous arms	This study
pMTLP_1,2-PD_cas-ΔeutT	pCB102 *ori, catP, ColE1 +tra, P_1,2-PD_-Cas9, P_araE_-sgRNA, eutT* homologous arms	This study
pMTL-cas9KI	pCB102 *ori, catP, ColE1 +tra, P_choline1_-Cas9, P_choline1_* homologous arms*, P_araE_-sgRNA*	This study
pMTL83151-P_arraE_	pCB102 *ori, catP, ColE1 +tra, P_araE_-sgRNA*	This study
pMTL-Δbdh::pdc	pCB102 *ori, catP, ColE1 +tra, P_araE_-sgRNA, bdh* homologous arms*, pdc*	This study
pMTL-3HB-KI	pCB102 *ori, catP, ColE1 +tra, P_araE_-sgRNA, 3hbdh* homologous arms*, CA_P_thl_, CB_thl, CB_ctfA, CB_ctfB*	This study
pMTL-cas9KO	pCB102 *ori, catP, ColE1 +tra, P_araE_-sgRNA, P_choline1_* homologous arms	This study

### Improved CRISPR/Cas9 tools for efficient metabolic engineering of *C. ljungdahlii*

**Fig 2:**
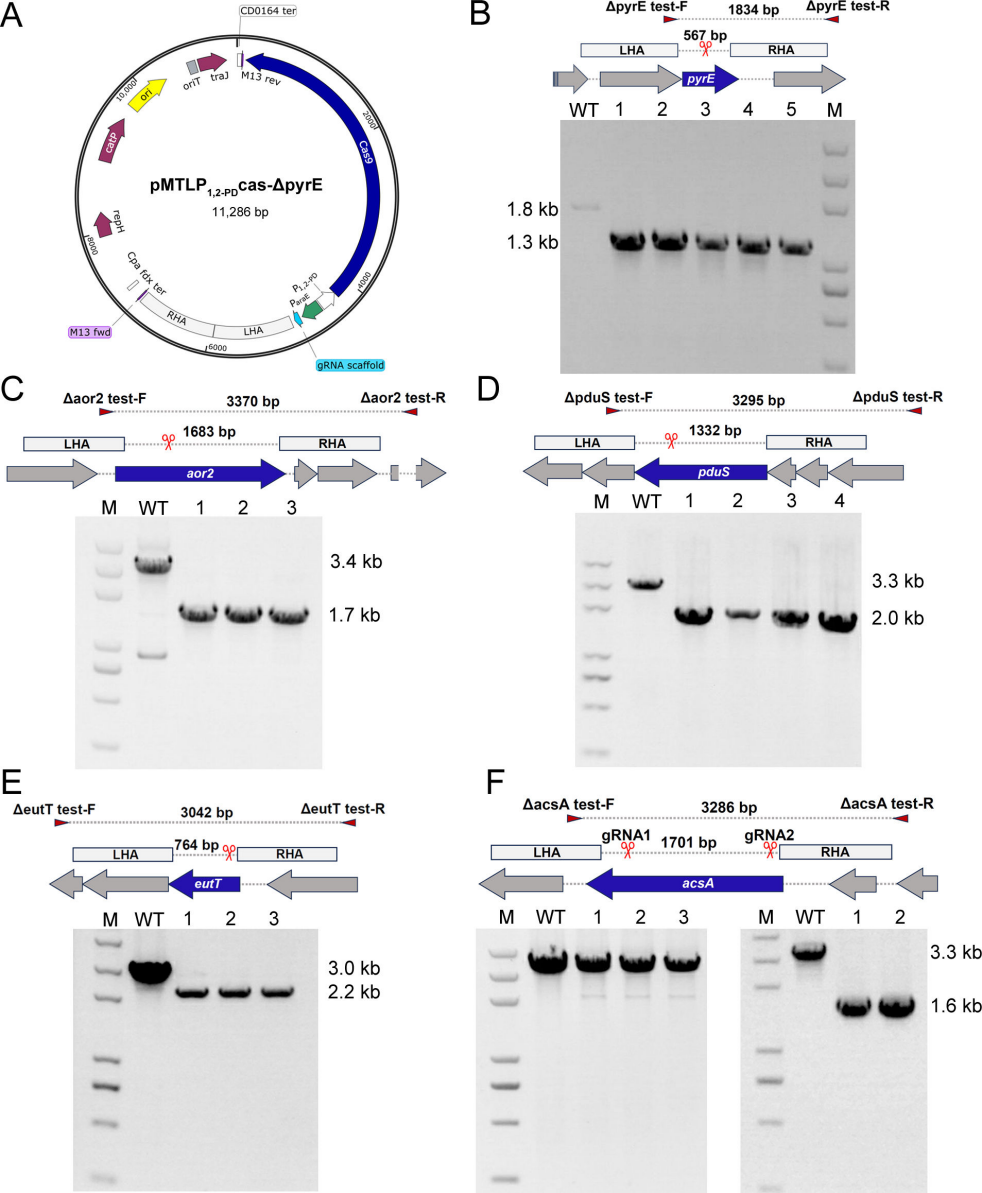
Gene editing of *C. ljungdahlii* based on the modified all-in-one CRISPR/Cas9 tool. (A) Construction of the plasmid pMTLP_1,2-PD_cas-ΔpyrE for *pryE* deletion. The genes *pryE* (B), *aor2* (C), *pduS* (D), *eutT* (E), and *acsA* (F) were targeted and deleted. The Cas9 cleavage sites were indicated using scissors. The primers for mutants screening were indicated using triangles. PCR amplification using specific primers and further sequencing of the PCR products confirmed the desired gene deletion. WT: wild-type strain.

The strong inducibility of P_1,2-PD_ made it an ideal candidate for genetic engineering. Based on the CRISPR/Cas9 gene editing system established by Huang et al., the promoter P_thl_ controlling the expression of *cas9* was replaced with P_1,2-PD_ (10) (Fig. 2A). This substitution enabled the efficient construction and propagation of gene deletion plasmids in *E. coli*, offering immediate results. To assess the editing efficiency of the modified system, four genes, namely, *pyrE* (CLJU_RS17570, encoding an orotate phosphoribosyltransferase), *pduS* (CLJU_RS19590, Table 1), *aor2* (CLJU_RS09915, encoding an aldehyde:ferredoxin oxidoreductase), and *eutT* (CLJU_RS19610, Table 1), were individually targeted for gene knockout (Table 2). The presence of gene deletion mutants in the transformant colonies was confirmed by colony PCR and further sequencing. All the four genes were deleted with 100% efficiency (Fig. 2B through E). Notably, we employed two gRNAs targeting the *acsA* gene (CLJU_RS18495) for gene deletion. One gRNA demonstrated an editing efficiency of 100%, whereas the other resulted in no edited cells among the three clones tested (Fig. 2F). This observation highlighted the importance of gRNA targeting efficiency for successful gene editing.

**Fig 3:**
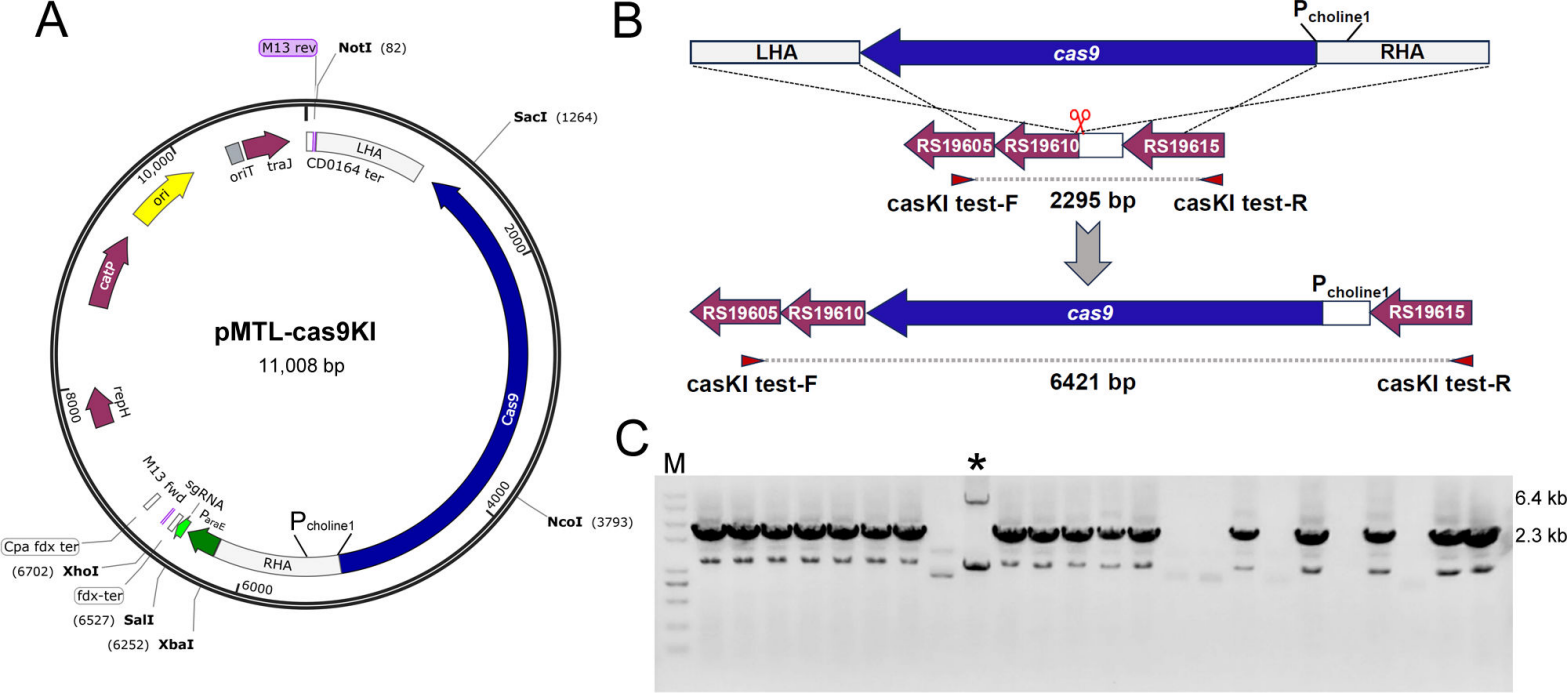
Integration of the *cas9* gene into the GRM1 loci under the control of P_choline1_. (A) Design of the plasmid pMTL-cas9KI for *cas9* gene integration. (B) Schematic illustration of the process for integrating the *cas9* gene into the GRM1 loci of *C. ljungdahlii* using the pMTL-cas9KI plasmid. (C) PCR screening of the *cas9* integration. The wild-type strain displayed 2.3 kbp bands, whereas the *cas9* integration strain showed 6.4 kbp bands. WT: wild-type strain.

Furthermore, to address the issue of the large sizes of all-in-one plasmids and facilitate gene insertion, the *cas9* gene was integrated into the genome under the tight control of P_choline1_. For the *cas9* integration plasmid, the gRNA targeting the P_choline1_ was under the control of the P_araE_ promoter (10). The *cas9* gene as part of the donor DNA was cloned between the relevant LHA and RHA (Fig. 3A). Additionally, to prevent the cleavage of the donor DNA by Cas9, the RBS of the P_thl_ instead of the native RBS of P_choline1_ was used. The resulting plasmid pMTL-cas9KI (Table 2) was transformed into *C. ljungdahlii,* and the expression of *cas9* was subsequently induced by supplementation of choline. The integration of *cas9* in the transformant colonies was verified through PCR, using one primer that flanked the LHA and another that bound the RHA. However, out of 24 tested colonies, only one was identified as a *cas9* integration mutant (Fig. 3B and C). We hypothesized that this low integration rate may be attributed to either insufficient expression of *cas9* or poor targeting efficiency of the gRNA. In any case, the positive recombinant was re-inoculated into the YTF medium without antibiotics for two consecutive subcultures to potentially eliminate the plasmid. The resulting recombinant strain CL-cas9KI (Table 2) was then inoculated into DSMZ 879 medium with either 5 g/L of fructose or choline as the carbon source. The transcription of *cas9* was compared under these two conditions by quantitative reverse transcription PCR (qRT-PCR), revealing that the expression of *cas9* was induced approximately 12 times in the presence of choline (Fig. S2).

### The integrated *cas9* conducted efficient gene editing in *C. ljungdahlii*

**Fig 4:**
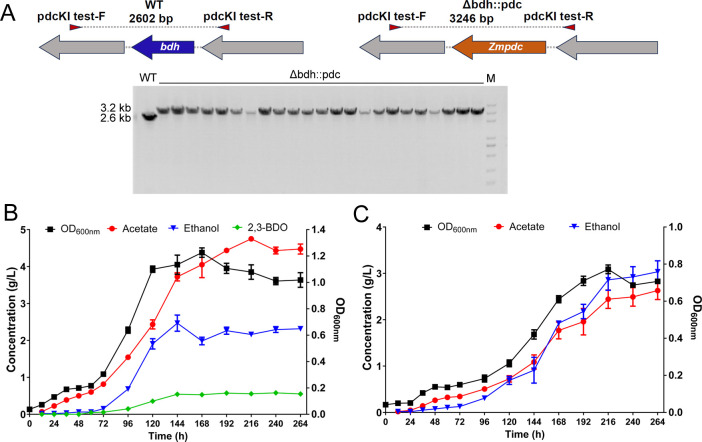
Growth and product profiles of *C. ljungdahlii* strains grown on CO:CO_2_ (vol/vol, 80/20). (A) PCR screening of the *Δbdh::pdc* strain. The wild-type strain displayed a 2.6 kbp band, whereas the recombinant strain showed 3.2 kbp bands. WT: wild-type strain. Growth and product profiles of the wild-type (B) and *Δbdh::pdc* recombinant strain (C) are shown.

The efficiency of the integrated *cas9* was evaluated through gene integration into the *C. ljungdahlii* genome. The primary fermentation products of *C. ljungdahlii* grown on syngas or CO are ethanol, acetic acid, and 2,3-butanediol. As the predominant byproduct, 2,3-butanediol constitutes approximately 25% of the total alcohol output and competitively inhibits ethanol production (25). To enhance the specificity of ethanol synthesis, the gene (CLJU_RS11425) encoding a 2,3-butanediol dehydrogenase was substituted with a pyruvate decarboxylase gene (*Zmpdc*, locus_tag: ZZ6_1712) from *Zymomonas mobilis* subsp. mobilis ATCC 29191. The pyruvate decarboxylase encoded by *Zmpdc* catalyzes the conversion of pyruvate to acetaldehyde, thereby reducing the precursors for 2,3-butanediol synthesis. The plasmid containing the gRNA cassette targeting CLJU_RS11425 and the donor DNA for recombination was introduced into the CL-cas9KI strain. Following induction with choline, the efficiency of the gene editing was evaluated via colony PCR. Given the low efficiency of *cas9* integration, a total of 23 clones were screened for the *pdc* insertion. Interestingly, the efficiency achieved was 100%, indicating that P_choline1_ mediated sufficient expression of *cas9* (Fig. 4A). Subsequent analysis of the growth and products of the recombinant strain indicated that the strain cultured in serum bottles no longer produced 2,3-butanediol when grown on CO (Fig. 4B and C).

**Fig 5:**
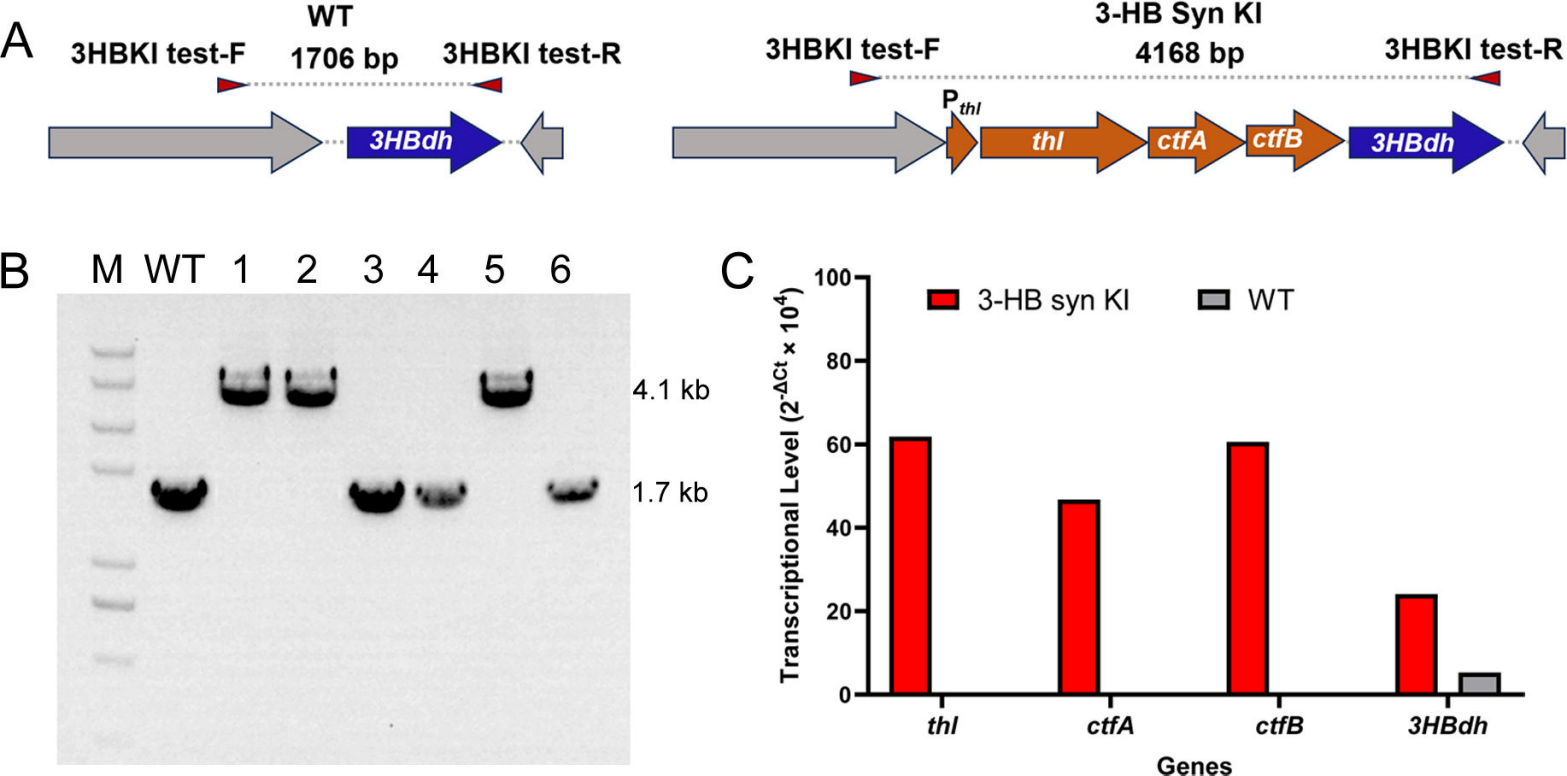
Genomic insertion of a 2.5 kbp artificial 3-hydroxybutyric acid (3-HB) synthesis pathway. (A) Gene organization of the wild-type and 3-HB synthesis pathway knock-in (3-HB Syn KI) strain (B) PCR screening of the 3-HB Syn KI strain. The wild-type strain displayed a 1.7 kbp band, whereas the recombinant strain showed 4.1 kbp bands. WT: wild-type strain. (C) Expression of the genes *thl*, *ctfA*, *ctfB*, and *3HBdh* was analyzed by qRT-PCR. The transcriptional level of the genes is represented as 2^-ΔCt^ ×10^4^. ΔCt = Ct (target gene) – Ct (16S rRNA gene).

The editing efficiency of the CRISPR/Cas9 system was further tested through the genomic insertion of a 2.5 kbp artificial 3-hydroxybutyric acid (3-HB) synthesis pathway. In a previous study by Jia et al., an endogenous 3-HB dehydrogenase encoded by CLJU_RS12255 was identified in *C. ljungdahlii*, which can convert acetoacetate into 3-HB (26). Consequently, we replaced the promoter of CLJU_RS12255 with a set of three genes (*thl*, *ctfA*, and *ctfB*) from *Clostridium beijerinckii*, all regulated by the P_thl_ from *C. acetobutylicum* (Fig. 5A). The enzymes encoded by the three genes are responsible for the conversion of acetyl-CoA to acetoacetate. The integration of the gene set into the target site resulted in an efficiency of 50%, with three out of six clones tested (Fig. 5B). qRT-PCR analysis revealed that the transcription level of CLJU_RS12255 in the recombinant strain was threefold higher than that in the wild-type strain (Fig. 5C). Additionally, the inserted genes *thl*, *ctfA*, and *ctfB* from *C. beijerinckii* were found to be highly transcribed. However, the recombinant strain did not exhibit any production of 3-HB (data not shown).

The system was further exemplified through genomic insertion of a hemoglobin gene from *Campylobacter jejuni* (Cj1586) into upstream of *cooS1* (CLJU_RS04490) and the insertion of *pduS* (CLJU_RS19590) and *pduT* (CLJU_RS19585) from GRM1 into the GRM3 gene cluster. Each instance resulted in an efficiency of 100% (Fig. S3).

In industrial applications, the removal of the *cas9* gene may be required. We showed that the *cas9* can be easily removed with an efficiency of 100% (6/6 clones tested, Fig. S4) by introducing a plasmid containing a gRNA cassette targeting the *cas9* gene along with the relevant homology arms.

### Conclusion

Inducible promoters allow for the transcription of target genes to be switched on and off in the presence or absence of inducers and have a broad range of applications. In this study, we identified two inducible promoters within the BMC gene clusters and utilized them to improve the CRISPR/Cas9 tools. Specifically, the P_1,2-PD_ was integrated into an all-in-one plasmid CRISPR/Cas9 tool to regulate the expression of the *cas9* gene, which facilitated the plasmid construction in *E. coli* and mediated desired gene deletion in *C. ljungdahlii* with 100% efficiency. The P_choline1_ was employed to tightly control the expression of a downstream genomically integrated *cas9* gene, which could efficiently mediate the gene insertion by introducing a plasmid containing a gRNA cassette along with the relevant homology arms. This improved tool offers an alternative to the all-in-one plasmid approach, potentially enhancing the transformation efficiency and playing a significant role in advancing genetic research and metabolic engineering of *C. ljungdahlii*.

## MATERIALS AND METHODS

### Strains and growth conditions

*Clostridium ljungdahlii* strains were routinely cultured anaerobically (N_2_-CO_2_-H_2_, 80:10:10) at 37°C in a modified DSMZ medium 879 (25). Genetic manipulations were done with YTF (yeast extract, 1%; Bacto Tryptone, 1.6%; sodium chloride, 0.4%; fructose, 0.5%; pH 6.0) liquid and solid media. For growth studies under heterotrophic conditions, 0.5% fructose was added to the modified DSMZ medium 879. When indicated, choline or 1,2-PD was supplemented as the sole or extra carbon source.

The fermentation of *C. ljungdahlii* strains was performed in 250-mL serum bottles with 50 mL working volume. The modified DSMZ medium 879 was supplemented with 5 g/L fructose or a headspace of a gas mixture (CO: CO_2_, 80: 20) as the carbon source. The bottles were incubated horizontally in a shaking incubator at 37°C and 200 rpm.

*Escherichia coli* was cultured at 37°C in Luria–Bertani (LB) medium (10 g L^−1^ NaCl, 10 g L^−1^ tryptone, and 5 g L^−1^ yeast extract) supplemented with appropriate antibiotics when required.

### Quantitative RT-PCR

Total RNA was isolated from the mid-exponential phase of *C. ljungdahlii* and reverse-transcribed into cDNAs using HiScript III RT SuperMix for qPCR (+gDNA wiper) (Vazyme, Nanjing, China). For each RNA sample, a negative RT (no addition of reverse transcriptase) was performed for the negative control. Primers for qPCR are listed in Table S1.

All qPCRs were performed using PerfectStart Green qPCR SuperMix (TransGen, Beijing, China) on LightCycler 480 (Roche, Basel, Switzerland), and the fold change of gene expression was calculated from three biological replicates using the 16S rRNA gene as a reference for normalization.

### Fermentation product analysis

Samples of *C. ljungdahlii* cultures were taken at indicated time points, and their absorbance at 600 nm was determined immediately. The cell pellets were subsequently removed by centrifugation at 10,000 × *g* for 5 minutes at 4°C, and the supernatants were subjected to high-performance liquid chromatography (HPLC) (Agilent 1200 Inﬁnity, Germany) for quantification. The 87 H column was used with 5 mM H_2_SO_4_ as the mobile phase.

### Plasmid construction

DNA sequences upstream of CLJU_RS05805, CLJU_RS19610, and CLJU_RS19635 were input into the iPro54-PseKNC tool to predict the promoter regions (http://lin-group.cn/server/iPro54-PseKNC) (27). To ensure the functionality of the predicted promoters, a probability score greater than 0.1 was used as a threshold to determine the 5′ nucleotides. The promoter regions were amplified via PCR using the primers listed in Table S1 and subsequently ligated with the linearized pMTL82254 vector obtained through digestion with NdeI (24) using the pEASY Seamless Cloning and Assembly Kit (TransGen, Beijing, China). The correct plasmids were selected in *E. coli* DH5α, resulting in the reporter plasmids pMTL82254-P_1,2-PD_, pMTL82254-P_choline1_, and pMTL82254-P_choline2_ (Table 2).

The plasmid pMTLP_1,2-PD_cas-ΔpyrE for *pyrE* deletion was assembled from the following fragments: the linear plasmid backbone obtained through double digestion of pMTLcas-pyrE (10) with NcoI and XbaI, the fragment of P_1,2-PD_ obtained via PCR amplification of *C. ljungdahlii* genome using primers P_1,2-PD_-F2 and P_1,2-PD_-R2, and the fragment of cas9-1 obtained by PCR amplification of pMTLcas-pyrE using primers cas9-1F and cas-1R. The three fragments were assembled using the pEASY Seamless Cloning and Assembly Kit (TransGen). The plasmids for *pduS* and *acsA* deletion were constructed using pMTLP_1,2-PD_cas-ΔpyrE as the template. The other two plasmids for *aor2* and *eutT* deletion were constructed using pMTLP_1,2-PD_cas-ΔpduS as the template. The gRNA and homologous arms (HAs) were changed accordingly. Each 20-nt target sequence for gRNA targeting was evaluated using CRISPy-web (https://crispy.secondarymetabolites.org/#/input), and the sequence with fewer potential off-target sites was chosen.

The plasmid pMTL-cas9KI (Table 2) for *cas9* integration was constructed using pMTLcas-pyrE as the template (10). pMTLcas-pyrE was subjected to double digestion with XhoI/SalI, and the resulting vector backbone was subsequently assembled with the gRNA scaffold obtained by PCR amplification of pMTLP_1,2_-PDcas-ΔpyrE using primers cas9KI-gRNA-F and gRNA-R3, employing the pEASY Seamless Cloning and Assembly Kit (TransGen). This resulted in the plasmid pMTL-casKI-1. The fragments cas9-LHA and cas9-RHA were generated by PCR amplification of the *C. ljungdahlii* genome using primers cas-LHA-F/cas-LHA-R and cas-RHA-F/cas-RHA-R, respectively. The fragments cas9-L and cas9-R were obtained via PCR amplification of pMTLcas-pyrE using primers cas9-L-F/cas9-L-R and cas9-R-F/cas9-R-R, respectively. The cas9-LHA fragment was assembled with cas9-L using overlap extension PCR, and similarly, cas9-RHA was fused with cas9-R via overlap extension PCR. The resulting assembled fragments were then double-digested with the appropriate restriction enzymes and subsequently cloned into pMTL-cas9KI-1, yielding the plasmid pMTL-cas9KI.

The promoter of the gene CA_C1339 from *C. acetobutylicum*, P_araE_, was obtained via PCR amplification of pMTL-cas9KI using primers P_1339_-F/P_1339_-R. The fragment of P_araE_ was subsequently double-digested with XbaI/SalI and ligated into pMTL83151 (24) to generate the plasmid pMTL83151-P_arraE_.

The plasmid pMTL83151-Δbdh::pdc, designed for the insertion of *Zmpdc,* was assembled from the following fragments: the linear plasmid backbone obtained by double digestion of pMTL83151-P_arraE_ with SalI and XhoI, the gRNA cassette amplified from pMTLP_1,2-PD_cas-ΔpduS using primers bdh-sgRNA-F / gRNA-R2, the HAs flanking the open reading frame of *bdh* amplified from the *C. ljungdahlii* genome using the primers bdh-LHA-F/bdh-LHA-R and bdh-RHA-F/bdh-RHA-R, and the *Zmpdc* gene fragment amplified from a codon-optimized *pdc* gene (synthesized by BGI, China) using the primers pdc-F/pdc-R. The gRNA cassette was first assembled with the LHA fragment via overlap extension PCR, yielding the gRNA-LHA fragment. The final step involved assembly of the plasmid backbone with the gRNA-LHA, *pdc*, and RHA fragments using the pEASY Seamless Cloning and Assembly Kit (TransGen).

The plasmid for 3-HB synthesis pathway integration was constructed in a similar way. The gRNA and homologous arms were amplified using appropriate primers. The promoter P_thl_ was amplified from pMTLcas-pyrE using primers P_THL_-F/P_THL_-R. The fragments of genes *thl* and *ctfAB* were obtained by amplifying the *Clostridium beijerinckii* NCIMB 8052 genomic DNA using primers thl-F/thl-R and ctfAB-F/ctfAB-R, respectively. The sgRNA scaffold and LHA were assembled via overlap extension PCR. Similarly, the P_thl_ promoter was assembled with the *thl* gene, and the *ctfAB* was fused with RHA. Finally, the three fragments were assembled with the pMTL83151-P_arraE_ backbone using the pEASY Seamless Cloning and Assembly Kit to yield the plasmid pMTL-3HB-KI (Table 2).

### Recombinant strain construction

The plasmids were introduced into *C. ljungdahlii* by electroporation. The electrocompetent cells were prepared according to the previously reported protocol (28). The electroporation was carried out as follows: 200 µL of electrocompetent cells was electroporated with 3 ~ 5 µg of plasmid DNA in a 2-mm cuvette (1.0 kV, 200 Ω, and 50 µF). The cells were recovered in 5 mL YTF medium for 12 hours and then plated onto YTF agar plates supplemented with 5 µg/mL of clarithromycin or 5 µg/mL thiamphenicol at 37°C for approximately 3–4 days.

Single colonies were inoculated into 5 mL 879 medium containing 3 g/L 1,2-PD or 5 g/L choline chloride and incubated at 37°C for 48 hours. The culture was streaked onto YTF agar plates, and the desired gene edition in the colonies was screened via PCR using corresponding specific primers (Table S1). The presence of possible line-like artifacts in the agarose gel pictures is due to image quality issues (see original files in the supplemental material). Finally, the correctness of the recombinant strains was confirmed by sequencing. All the correct mutants were serially sub-cultured in the YTF liquid medium without antibiotics to eliminate the plasmids.

### Promoter activity assays

Strains of *C. ljungdahlii* carrying pMTL82254-P_1,2-PD_, pMTL82254-P_choline1_, or pMTL82254-P_choline2_ were inoculated into the DSMZ medium 879 supplemented with 3 g/L 1,2-PD or 5 g/L choline chloride at an initial OD_600nm_ of 0.01. Cultures grown in media without the respective inducer served as negative controls. Samples were collected at 12 hours, 36 hours, and 60 hours post-inoculation, followed by centrifugation to harvest the cells.

The activity of CatP was determined using a modified DTNB (5,5′-dithiobis-2-nitro-benzoic acid) method, which is an adaptation of the approach by Cañadas et al. (13). The cells were first resuspended in a 0.1 M Tris-HCl buffer (pH 8.0), followed by sonication to extract the crude enzyme solution. The total protein of the crude enzyme solution was quantified using the Bradford Protein Assay Kit (Sangon, China). A 150-µL detection mixture was prepared in a 96-well clear-bottom plate, consisting of 94 mM Tris-HCl buffer (pH 8.0), 0.19 mM acetyl coenzyme A, 0.1666 mM DTNB, and 5 mg/mL chloramphenicol. The mixture was then mixed with 10 µL of the properly diluted crude enzyme solution and 40 µL of 0.1 M Tris-HCl buffer (pH 8.0). The activity of CatP was assayed at 25°C by continuously monitoring the absorbance at 412 nm for 1 minute. The rate of increase of absorption (*k*_A412_) was used to calculate chloramphenicol acetyltransferase (CAT) activity (U/mg) using the following equation, where 0.2 is the total volume (in mL) of the assay, *df* is the dilution factor, 13.6 is the micromolar extinction coefficient (A/(mM·cm)) for TNB at 412 nm, 0.01 is the volume (in mL) of the cell lysate used, 0.552 is the thickness (in cm) of the liquid in the well, and *c* is the protein concentration (in mg/mL) of the crude enzyme solution.


$$U/mg=\frac{\left(k_{A412})(0.2)(df\right)}{\left(13.6)(0.01)(0.552)(c\right)}$$

## Data Availability

All relevant data are within the article and the supplemental material.

## SUPPLEMENTAL MATERIAL

Supplemental material aem.02183-24-s0001.pdf Table S1; Figures S1 to S4.

ASM does not own the copyrights to Supplemental Material that may be linked to, or accessed through, an article. The authors have granted ASM a non-exclusive, world-wide license to publish the Supplemental Material files. Please contact the corresponding author directly for reuse.
