# Supplementary material for: Inducible promoters of bacterial microcompartments improve the CRISPR/Cas9 tools for efficient metabolic engineering of Clostridium ljungdahlii
Source: Appl Environ Microbiol. 2025 Mar 26;91(4):e02183-24. doi: 10.1128/aem.02183-24 (PMC12016505; doi:10.1128/aem.02183-24)
Supplement: Supplemental material — Table S1; Figures S1 to S4. [file aem.02183-24-s0001.pdf]

Supplemental Information for

Inducible Promoters of Bacterial Microcompartments Improve the CRISPR/Cas9 Tools for Efficient Metabolic Engineering of *Clostridium ljungdahlii*

Jun-Zhe Zhang[a,c,e], Yu-Zhen Li[a,c,e], Zhi-Ning Xi[a,c], Yue Zhang[b], Zi-Yong Liu[a,c], Xiao-Qing Ma[a,c,#], Fu-Li Li[a,c,d,#]

[a]Key Laboratory of Photoelectric Conversion and Utilization of Solar Energy, Qingdao Institute of Bioenergy and Bioprocess Technology, Chinese Academy of Sciences, Qingdao 266101, China;

[b]Haide College, Ocean University of China, Qingdao 266000, China;

[c]Shandong C1 Refinery Engineering Research Center, Qingdao New Energy Shandong Laboratory, Qingdao Institute of Bioenergy and Bioprocess Technology, Chinese Academy of Sciences, Qingdao, China;

[d]Shandong Energy Institute, Qingdao 266101, China.

[e]University of Chinese Academy of Sciences, Beijing 100049, China;

#Address correspondence to Xiao-Qing Ma, maxq@qibebt.ac.cn, or Fu-Li Li, lifl@qibebt.ac.cn.

This file includes:

Supplemental Materials and Methods

Table S1

Figure S1-S4

Orignal files of Figre 3C, 4A, and 5B

Supplemental Materials and Methods

Substrate and product analysis. The residual fructose and 1,2-PD as well as generated acetate, ethanol, n-propanol, isopropanol, and propanoate were detected by Agilent 7890A/5975C GC-MS, and then subjected to high-performance liquid chromatography (HPLC) system (Agilent 1200 Infinity, Germany) for quantification. The 87-H column was used with 5 mM H_2SO_4 as the mobile phase.

The residual choline and generated trimethylamine (TMA) were analyzed by high-performance anion-exchange chromatography with pulsed amperometric detection (HPAEC-PAD) using Dionex ICS-3000 (Sunnyvate, USA).

Transmission electron microscopy. *C. ljungdahlii* cells grown on choline or 1,2-PD were collected at mid-log phase and fixed with 2.5% glutaraldehyde for 2 h at room temperature. The cells were washed three times with 0.1 M PBS and further fixed with 1% osmium tetraoxide for 1 h. After washing three times with PBS, the cells were dehydrated in a serial increasing concentrations of acetone. Samples were embedded in Epson 812 resin medium and polymerized at 70°C for 24 h. Thin sections were successively stained with 1% uranyl acetate and lead citrate, and observed in a JEM-F200 transmission electron microscope at 200 kv.

Table S1 Primers used in this study.

Primers	Sequence (5'-3')
Primers for qPCR	
q-16S-F	TGGTAGTCCACGCCGTAAACGA
q-16S-R	GGGCCCCCGTCAATTCCTTT
q-cas9-F	ATTTTGCGGTTGCTTTGCCT
q-cas9-R	CCGTCGTTGGAACTGCTTTG
q-RS05800-F	GGCTCTTGCAATATGTGGAACTAA
q-RS05800-R	ATCCTGCTATCACCGGGCTAT
q-RS05805-F	ACAGACAGGAGCAACACCAG
q-RS05805-R	TGGTCCCATTGCATCTCTTCC
q-RS05810-F	AGACGATCTGGTGGTGGAAT
q-RS05810-R	TGCTGCGGTTTCAATAGCTG
q-RS05815-F	CCTACTTCCATGGCAATTTGTGT
q-RS05815-R	TGGGAGTGACATTTACCCAGT
q-RS05820-F	ATGCGTTCAGCAGCTTCCTT
q-RS05820-R	GTGTTGGGCGGACAGGTATT
q-RS05825-F	TGATGCGGGACATGCAGAAC
q-RS05825-R	CCTGCTGGTGCTCCTACAAC
q-RS05830-F	TACAGATGCGCTGGCTGAAA
q-RS05830-R	GCCATACCAGCCATGCAAGA
q-RS05835-F	TGCCAGCTTTGGCTATAGGTT
q-RS05835-R	CCATCCTGCCAGCACACATA
q-RS05840-F	TGGGACAAGACTTACACCTGA
q-RS05840-R	TGCTTCTTCTCCACAGGTTG
q-RS05845-F	GGTTGGAGGAACTTGCTGTCT
q-RS05845-R	CCTGCGTACAGCTAAGTGCTA
q-RS05850-F	TGAGGCGTATGGGTGCTAATG
q-RS05850-R	CTACTGTGACACCAACTGCCT
q-RS05855-F	TGGGCAACAAGAAAAGCAGA
q-RS05855-R	TCTCCAATTCCGGCACTGAT
q-RS05860-F	ACTCCATCAGCCAAAGATATAGCA
q-RS05860-R	TCACAAGGAGTTTCCACTTCACA
q-RS05865-F	GCTATGCTCAAGGCAGCAGA
q-RS05865-R	TTCACAGCACCCACATCACC
q-RS05870-F	GCGTTGGTGCTGGTAACTCT
q-RS05870-R	CAGACTGCTCGGATGCACAA
q-RS05875-F	GGGTGCATGATGGACAGAC
q-RS05875-R	GAGTTGCCAACACACATTGC
q-RS05880-F	TGTAGGAGCAGTAAAGGCAGC

q-RS05880-R	TTCAACGTGTGGACGAGGAAT
q-RS12245-F	GGCCATGAAGCTGTAGGTGA
q-RS12245-R	TCTCCAGTCAGGTGTTGTGC
q-RS19540-F	ACAAGTGGTACAGGCTCTGAAG
q-RS19540-R	TGCTACAATACGCTGAGGCA
q-RS19545-F	TGTATTGCCCGTGTGGCTTAT
q-RS19545-R	CCAATGGTGTGAAGCTGGTTAC
q-RS19550-F	TCTGCTCACTCGGACTTTTC
q-RS19550-R	AGCTGCGGAGTCTAACATCT
q-RS19580-F	TCCACTATTTCTAGCCGCATCTT
q-RS19580-R	AACTGCAGAAAAGCAGGGTCA
q-RS19585-F	TGCATCAGCAGCAATGACC
q-RS19585-R	CGGCAGTATTCCCTGCAATC
q-RS19590-F	GGATAAAGCCCTGCTGGACA
q-RS19590-R	ACCCCACAAGATGATGAGAGC
q-RS19610-F	GTGTAAGGACCATCGTCAAACAT
q-RS19610-R	ACCGGGAACAAGACTTACGC
q-RS19615-F	AGAGACACAAGCACCGCAAT
q-RS19615-R	ATGGACCGGGAGTAAGGACA
q-RS19620-F	GGGTTTGAATCTCCACCACCA
q-RS19620-R	AGACCAGTACCGTGAAGCAG
q-RS19625-F	CCATATCCGACTTTGCGGAC
q-RS19625-R	TACGCCTGCTTCCTTTGGTA
q-RS19630-F	AGCTGCTTTGCAAGCTCCTA
q-RS19630-R	AGAGGCTGCAGATGCAATGT
q-RS19635-F	TCCTGCTTCCACAGATGCTC
q-RS19635-R	AAGGCTGCTGACGTTGAACT
q-thl-F	TTGGAGCATCAGGTGCAAGA
q-thl-R	CCCATTCCGCCACCTATACA
q-ctfA-F	ATGGCAATGGCTGCAAAAACA
q-ctfA-R	AGCCGCCTCCTTAACGATATAA
q-ctfB-F	GTTGCTGTTCTTGGTGCTCT
q-ctfB-R	ATCCATAGCGCCACCCATAC
q-3HBdh-F	GCCCAATGTCTAGCTGGGAT
q-3HBdh-R	TGGCATTAGCACACCCATGA
Primers for pMTL82254-$P_{1,2\text{-}PD}$ construct	
$P_{1,2\text{-}PD}$-F	GACCGCGGCCGCTGTATCCACTATATAATTTAGATTTCTATTTTGCAGA
$P_{1,2\text{-}PD}$-R	CTATTTTTATCAATTTTTTCAAATACCATATAAACAACCCCCTTAATTAATT
Primers for pMTL82254-$P_{choline1}$ construct	
$P_{choline1}$-F	GACCGCGGCCGCTGTATCCAATGATTTGGATAGAATTGAAGGCTGGA
$P_{choline1}$-R	CTATTTTTATCAATTTTTTCAAATACCATATTGGCCCCCCTTTACTTTTATT

Primers for pMTL82254-$P_{choline2}$ construct	
$P_{choline2}$-F	GACCGCGGCCGCTGTATCCAATGAAACCACTTAATTATGCTATTTTAAAACAC
$P_{choline2}$-R	CCAACTATTTTTATCAATTTTTTCAAATACCATATCATTACCCTCCTCCTCTATACG
Primers for pMTL$P_{1,2\text{-}PD}$cas-ΔpyrE construct	
$P_{1,2\text{-}PD}$-F2	GCCTATTGAGTATTTCTTATCCATTTAAACAACCCCCTTAATTAATTTTA
$P_{1,2\text{-}PD}$-R2	ACTAAATATAAATCTAGATTTTTAACAAAACTATATAATTTAGATTTCTATTTTGC
cas9-1F	GCACCTTTATCGACAACTTCTTCA
cas9-1R	ATGGATAAGAAATACTCAATAGGCTTA
Primers for pMTL$P_{1,2\text{-}PD}$cas-ΔpduS construct	
pduS-gRNA-F	CTTAAGGAGGAGTTTTCGTCGACCTGGCAATAAGACGCTTACTGTTTTAGAGCTAGAAA
gRNA-R1	ATAAAAATAAGAAGCCTGCAAATGCAGGCTTCTTATTTTTATAAAAAAAGCACCGACTC
pduS-LHA-F	CCTGCATTTGCAGGCTTCTTATTTTTATCCAACGGCTGCTAATAAAGTTCTTAGA
pduS-LHA-R	TTATTCTACATTGCAGTCTTCATCAATAA
pduS-RHA-F	AAGACTGCAATGTAGAATAAATGTAAGGAGGGGATAATATGTCACAAGCA
pduS-RHA-R	CATGTCTGCAGGCCTCGAGTTCATACTTTACTGAACTACCACGAA
Primers for pMTL$P_{1,2\text{-}PD}$cas-Δaor2 construct	
aor2-gRNA-F	CTTAAGGAGGAGTTTTCGTCGACTTTTATGCATAGGACCAGCTGTTTTAGAGCTAGAAA
gRNA-R2	CCGTTGGTTCATCAAGCAC
aor2-LHA-F	GTGCTTGATGAACCAACGGTGTATTGATAACTGGTGAGAGTGGTAC
aor2-LHA-R	ATACCTTACCCTTATATCCGTACATAAAA
aor2-RHA-F	ATGTACGGATATAAGGGTAAGGTATAGTTAGATGTACTACTACCTGAATATTATTCAGT
aor2-RHA-R	TTGCATGTCTGCAGGCCTCGAGATTTCATACTCACTAGAGAGAAGATCCA
Primers for pMTL$P_{1,2\text{-}PD}$cas-ΔeutT construct	
eutT-gRNA-F	CTTAAGGAGGAGTTTTCGTCGACGATAGAAATTGACGTGCTCCGTTTTAGAGCTAGAAA
gRNA-R2	CCGTTGGTTCATCAAGCAC
eutT-LHA-F	GTGCTTGATGAACCAACGGATTGAATCTTTATTAACATTAGCACCCA
eutT-LHA-R	TGTTTATTTTTAAAATAAAAGTAATGGAGGTACAAAATGTCAGAGAAAAATTTG
eutT-RHA-F	CATTTTGTACCTCCATTACTTTTATTTTAAAAATAAACATTTTTTAGGGCTCC
eutT-RHA-R	ATGTCTGCAGGCCTCGAGTTGCGGTGCTTGTGTCTCTG
Primers for pMTL$P_{1,2\text{-}PD}$cas-ΔacsA construct	
acsA-gRNA1-F	CTTAAGGAGGAGTTTTCGTCGACAATGATGTAATTGTTGTTGTGTTTTAGAGCTAGAAA
acsA-gRNA2-F	CTTAAGGAGGAGTTTTCGTCGACGGCACTGGACTTACTCTACAGTTTTAGAGCTAGAAA
gRNA-R1	ATAAAAATAAGAAGCCTGCAAATGCAGGCTTCTTATTTTTATAAAAAAAGCACCGACTC
acsA -LHA-F	CCTGCATTTGCAGGCTTCTTATTTTTATGACTCTATCAGAACTTCCAACTG
acsA -LHA-R	GACAAGGCAAAACAAGATGGGGACATTCTTACTAATAAGATGGAAGATTGG
acsA -RHA-F	CCCATCTTGTTTTGCCTTGTC
acsA -RHA-R	ATGTCTGCAGGCCTCGAGGTCTATCCAGTAGCAGATACAACAG
Primers for pMTL-cas9KI construct	
cas9KI-gRNA-F	CTTAAGGAGGAGTTTTCGTCGACTTTAAAATAAAAGTAAAGGGGTTTTAGAGCTAGAAA
gRNA-R3	CATGTCTGCAGGCCTCGAGATAAAAATAAGAAGCCTGCAAATGCAGGCTTCTTATTTTTATA A

cas-LHA-F	ACCGCGGCCGCGCTTCATCTCTCTAACAATATTGATTGC
cas-LHA-R	GAATTAGGGGGGCCAAAATGAAATTTAT
cas9-L-F	CATTTTGGCCCCCCTAATTCTCAGTCACCTCCTAGC
cas9-L-R	CATTTATTTACGTTGACGAATCTTGG
cas-RHA-F	CTTATCCATAGATTTGGCCTCCTTTACTTTTATTTTAAAAATAAACATTTTTTAGGGC
cas-RHA-R	ATAAATCTAGATTTTTAACAAAAAAAATTCATGCATTAATTGCGG
cas9-R-F	ACTTCTTCAAAATTCCATGGGGTAAT
cas9-R-R	AGGAGGCCAAATCTATGGATAAGAAATACTCAATAGGCTTAGAT
Primers for pMTL-P_{araE}	
P_{1339}-F	AAATCTAGATTTATATTTAGTCCCTTG
P_{1339}-R	GGGGTCGACGAAAACTCCTCCTTAAG
Primers for pMTL-Δbdh::pdc construct	
bdh-gRNA-F	CTTAAGGAGGAGTTTTCGTCGACAGAAGGAAATTCATGTCTGTGTTTTAGAGCTAGAAA
gRNA-R2	CCGTTGGTTCATCAAGCAC
bdh-LHA-F	GTGCTTGATGAACCAACGGCTCCTTTAAATCCATTTGCTTTAAACAC
bdh-LHA-R	CCTTATTGTAAAAAGTACTCATAGAATTG
pdc-F	CAATTCTATGAGTACTTTTTACAATAAGGCTAAAGAAGCTTATTTACAGGTTTTCTACT
pdc-R	ATGAGTTACACTGTAGGCACTTAC
bdh-RHA-F	GTAAGTGCCTACAGTGTAACTCATAATTATCTCTCCTTTTTTATAATAGTATGGCAAT
bdh-RHA-R	TGTCTGCAGGCCTCGAGCACAATTATAGTGAAAGATGTGAAGGC
Primers for pMTL-3HB-KI construct	
gRNA-F	CTTAAGGAGGAGTTTTCGTCGACTGGGAAGATTTACTTTGCCTGTTTTAGAGCTAGAAA
gRNA-R2	CCGTTGGTTCATCAAGCAC
LHA-F	GTGCTTGATGAACCAACGGGAAAAAATGATAAATTTAAAAGAACAGTTA
LHA-R	AAAAACAACAATTTTTCTTAAAATACTGTTGCTTTTTACAACAGTGTTG
P_{THL}-F	CAACAGTATTTTAAGAAAAATTGTTGTTTTTATAATTAAGTTGTTAGAGAAAACGTATA
P_{THL}-R	TCTAACTAACCTCCTAAATTTTGATACGG
thl-F	CCGTATCAAAATTTAGGAGGTTAGTTAGAATGAGAGAGGTAGTTATTGTAAGTG
thl-R	TATTCATTTTAATATCTCCCTTCTTAAATTCTTTCAACGATAAGTGCTGTTC
ctfAB-F	AATTTAAGAAGGGAGATATTAAAATGAATAAATTAGTAAAATTAACAGATTTAAAGCG
ctfAB-R	CTTCCCATTTTTGTCCTCCTTATTATTCATATATCCATAATCTTTAAGTTATCTGGAAT
RHA-F	AAGGAGGACAAAAATGGGAAGATTTACATTACCAAGGGATATTTACTTTGGTG
RHA-R	ATGTCTGCAGGCCTCGAGCGTTTTCGCTTATACCATAATCTTTTATG
Primers for pMTL-cas9KO construct	
cas-gRNA-F	CTTAAGGAGGAGTTTTCGTCGACAGATGATTGAGGAAAGACTTGTTTTAGAGCTAGAAA
gRNA-R2	CCGTTGGTTCATCAAGCAC
HA-F	GTGCTTGATGAACCAACGGTCCAATAGTATCGCTGCACAATG
HA-R	TGCATGTCTGCAGGCCTCGAGATGAGTAATGCGAAGGCAGCAATG
Primers for mutant screening	
ΔpyrE test-F	CCTTCTATATTCTGAACCATAACATGA
ΔpyrE test-R	GGCGGGAGCTTATGCAATTCAAGTA

ΔpduS test-F	TTAGAACAATGATGCCAATAATCTT
ΔpduS test-R	GTTCTGCGACCAACTTGTGAAAGATTTT
Δaor2 test-F	GGCAATGGGGATTGGAAGAA
Δaor2 test-R	CCTGATGTGGGTCAATAAGGAA
ΔeutT test-F	CCTGCGTACAGCTAAGTGCTA
ΔeutT test-R	GGTAATGGACAAATGCAGTTCAAT
ΔacsA test-F	CCCTTATAATTTATGTATTTGTAGATAA
ΔacsA test-R	CAATCATCATTGCGAACACTAAAC
casKI test-F	AACTGCCCCTGAATCCAAATCATC
casKI test-R	ATGAGTAATGCGAAGGCAGCAATG
pdcKI test-F	GGCATTCTGAGCCAGTTCTTTTAA
pdcKI test-R	GATGGCCCCTTTGGATTATTT
3HBKI test-F	GGCCTGGAAATGTTAGAGAAT
3HBKI test-R	AACTAGAAATTAACCTTTTGCCCA
casKO test-F	CCTGCGTACAGCTAAGTGCTA
casKO test-R	GGTAATGGACAAATGCAGTTCAAT

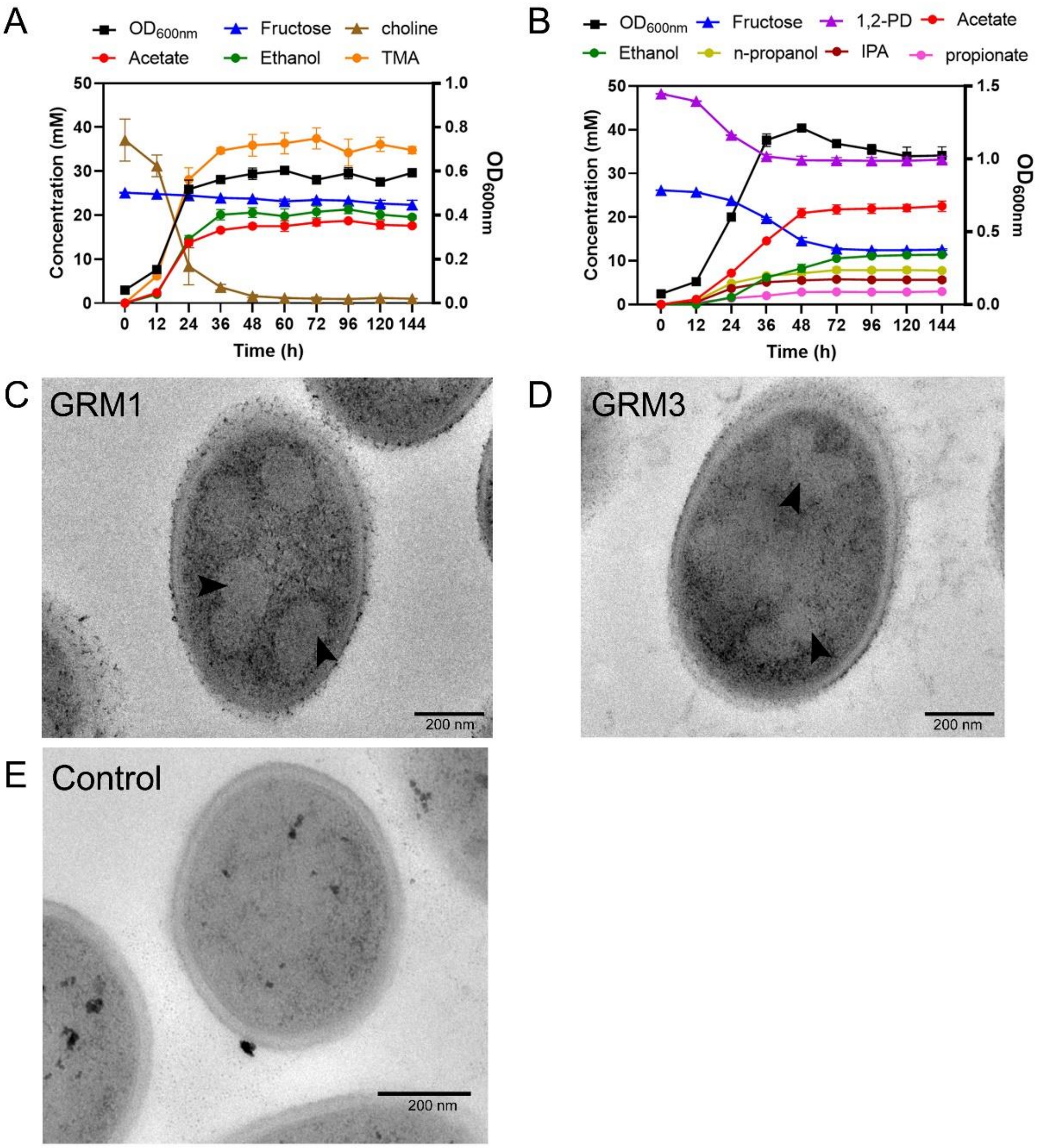

Figure S1 Two GRMs in *C. ljungdahlii* involved in choline and 1,2-PD metabolism. *C. ljungdahlii* utilized choline (A) and 1,2-PD (B) as carbon source. The substrate consumption and product production were monitored. The polyhedral structures hypothesized to be BMCs were induced by the presence of choline (C) and 1,2-PD (D), respectively. The subcellular structure indicated by arrows were presumed to be GRMs. The GRM1, approximately 100 nm in cross-section, exhibited irregular polyhedral shapes with distinct boundaries; the GRM3 appeared cotton-like without well-defined edges. (E) A cell image without BMCs. The cells were grown on fructose.

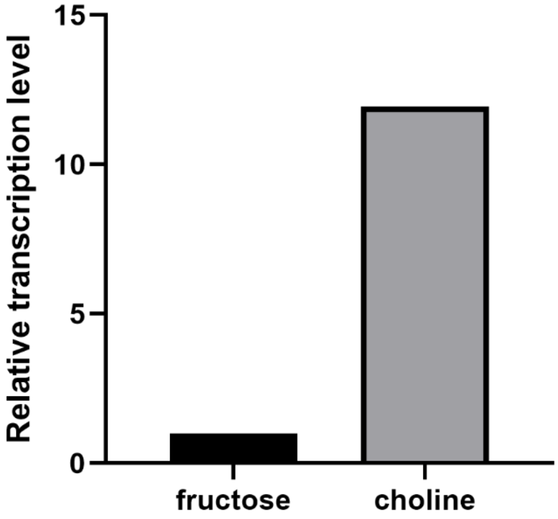

Figure S2 The qRT-PCR results depicted $\log_2$ fold change of *cas9* expressed by CL-cas9KI grown on choline relative to fructose.

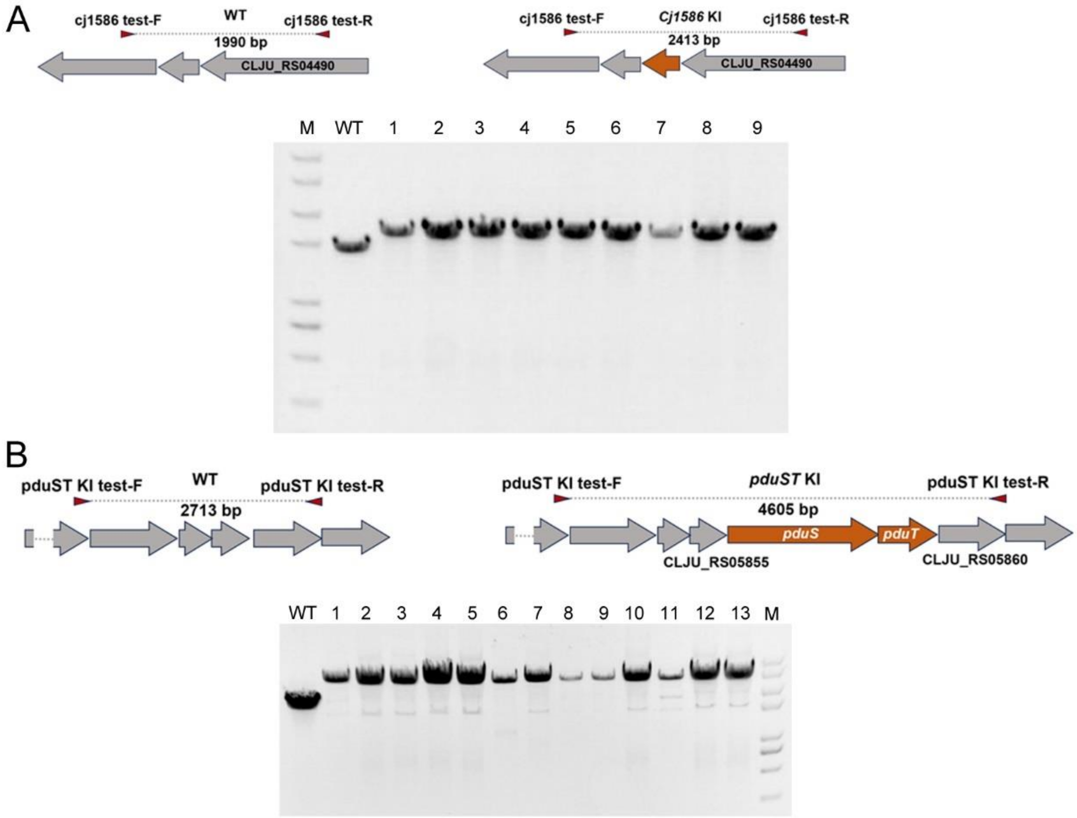

Figure S3 PCR screening of genomic insertion of the hemoglobin gene Cj1586 from *Campylobacter jejuni* (A) and *pduST* (CLJU_RS19590 and CLJU_RS19585) (B).

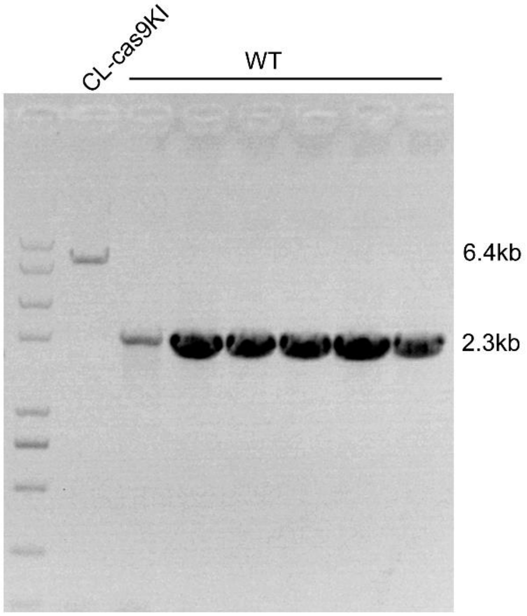

Figure S4 PCR screening of the *cas9* removal. The 6.4 kbp band represents the cas9 integration genotype, whereas the 2.3 kbp bands represent the wild-type genotype.

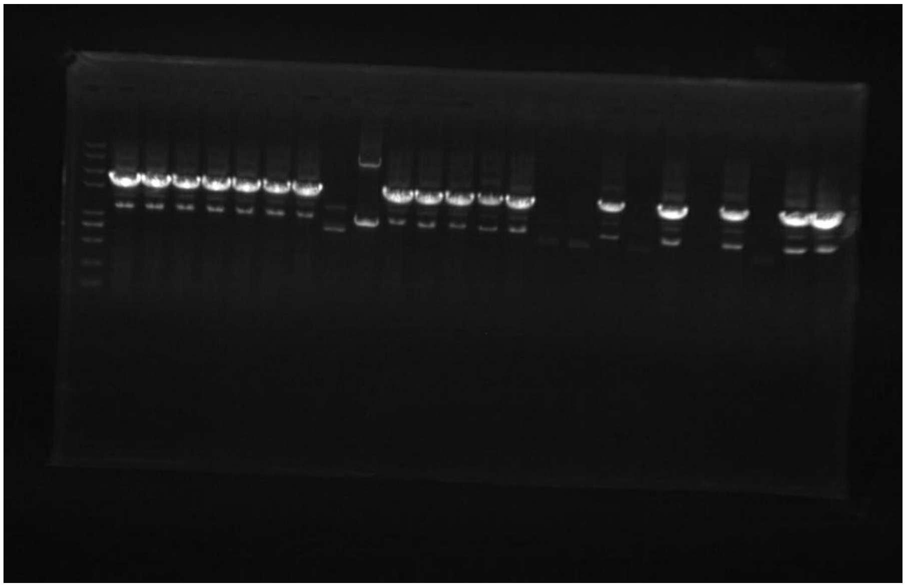

Original file of figure 3C

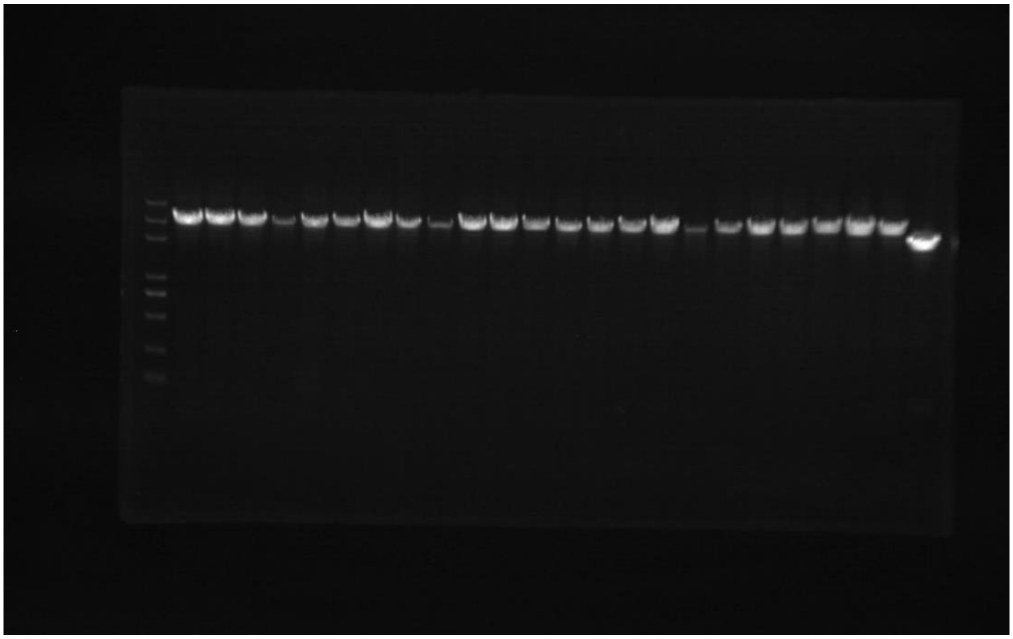

Original file of figure 4A

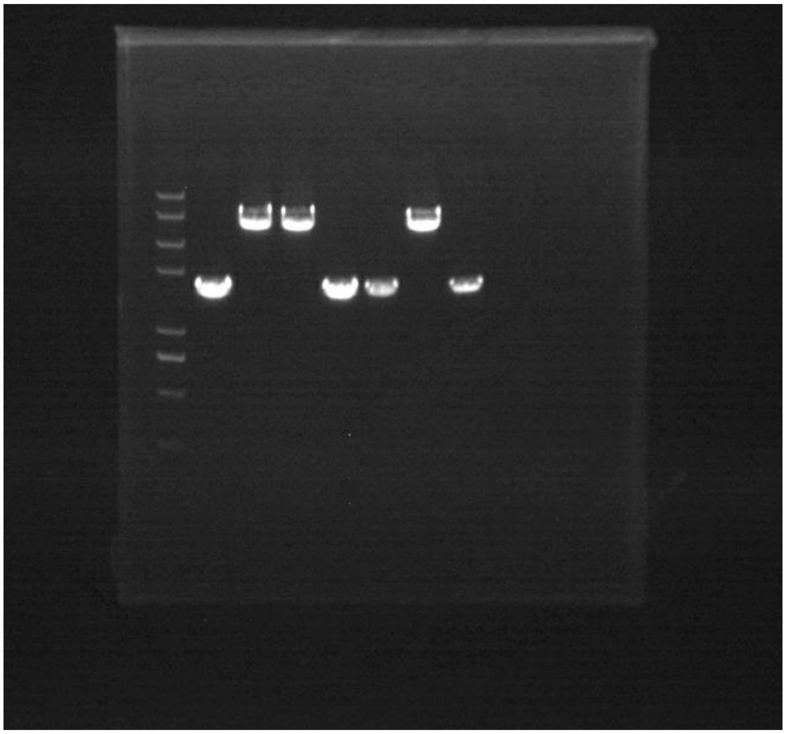

Original file of figure 5B
